# The Impact of Nanoparticle Coatings on the Color of Teeth Restored Using Dental Adhesives Augmented with Magnetic Nanoparticles

**DOI:** 10.3390/medicina61071289

**Published:** 2025-07-17

**Authors:** Carina Sonia Neagu, Andreea Codruta Novac, Cristian Zaharia, Meda-Lavinia Negrutiu, Izabell Craciunescu, Vlad Mircea Socoliuc, Catalin Nicolae Marin, Ionela-Amalia Bradu, Luminita Maria Nica, Marius Stef, Virgil-Florin Duma, Mihai Romînu, Cosmin Sinescu

**Affiliations:** 1 Department of Prosthetic Technology and Dental Materials, “Victor Babes” University of Medicine and Pharmacy Timisoara, 9 Revolutiei 1989 Ave., 300070 Timișoara, Romania; carina_neagu@yahoo.com (C.S.N.); cojocariu.andreea@umft.ro (A.C.N.); cristian.zaharia@umft.ro (C.Z.); negrutiu.meda@umft.ro (M.-L.N.); luminita.nica98@yahoo.ro (L.M.N.); rominu.mihai@umft.ro (M.R.); minosinescu@yahoo.com (C.S.); 2 Research Center in Dental Medicine Using Conventional and Alternative Technologies, “Victor Babes” University of Medicine and Pharmacy Timisoara, 9 Revolutiei 1989 Ave., 300070 Timișoara, Romania; 3 National Institute for Research and Development of Isotopic and Molecular Technologies, 67-103 Donat, 400293 Cluj-Napoca, Romania; izabell.craciunescu@itim-cj.ro; 4 Romanian Academy–Timisoara Branch, Center for Fundamental and Advanced Technical Research, Laboratory of Magnetic Fluids, 24 Mihai Viteazu Ave., 300223 Timișoara, Romania; vsocoliuc@gmail.com; 5 Research Center for Complex Fluids Systems Engineering, Polytechnic University of Timisoara, Mihai Viteazu Ave. No. 1, 300222 Timișoara, Romania; 6 Faculty of Physics, West University of Timișoara, 4 Vasile Parvan Ave., 300223 Timișoara, Romania; catalin.marin@e-uvt.ro (C.N.M.); marius.stef@e-uvt.ro (M.S.); 7 Research Centre for Thermal Analysis in Environmental Problems, West University of Timișoara, Pestalotzzi Street No. 16, 300115 Timișoara, Romania; ionela.bradu@e-uvt.ro; 8 ICAM—Advanced Environmental Research Institute, West University of Timișoara, Oituz Street No. 4C, 300086 Timișoara, Romania; 9 3OM Optomechatronics Group, Department of Measurements and Optical Electronics, Faculty of Electronics, Telecommunications, and Information Technology, Polytechnic University of Timisoara, 2 Vasile Parvan Ave., 300223 Timișoara, Romania

**Keywords:** dental adhesive, magnetic nanoparticles, adhesion, coatings, direct restorations, colorimetric analysis, dental spectrophotometer, esthetic impact, polymerization, magnetization

## Abstract

*Background and Objectives*: Dental adhesives augmented with magnetic nanoparticles (MNPs) have been proposed to prevent microleakages. MNPs dispersed in a dental adhesive reduce the thickness of the adhesive layer applied in a magnetic field and enhance the bond strength by favoring the penetration of the adhesive into dentinal tubules. However, the restoration’s color has been found to be affected by the MNPs. This study tests the hypothesis that MNP coating can alleviate the esthetic impact of magnetic dental adhesives. *Materials and Methods*: We synthesized Fe_3_O_4_ MNPs with silica coating (MNPs-SiO_2_), calcium-based coating (MNPs-Ca), and no coating. Their morphology was studied using transmission electron microscopy (TEM) and scanning electron microscopy (SEM). Their chemical composition was assessed by energy-dispersive X-ray spectroscopy (EDX), and magnetic properties were measured using a vibrating sample magnetometer. FTIR spectroscopy was used to evaluate the polymerization of the MNP-laden adhesive. We prepared cavities in molar phantoms divided in four groups (n = 15 each) restored using the same adhesive with different MNP contents: Group 0 (G0)—no MNPs, G1—MNPs-SiO_2_, G2—MNPs-Ca, and G3—uncoated MNPs. The restoration’s color was quantified in the CIELAB color space using a dental spectrophotometer. *Results*: MNPs-SiO_2_ were globular, whereas MNPs-Ca had a cubic morphology. The SiO_2_ layer was 73.1 nm ± 9.9 nm thick; the Ca(OH)_2_ layer was 19.97 nm ± 2.27 nm thick. The saturation magnetization was 18.6 emu/g for MNPs-SiO_2_, 1.0 emu/g for MNPs-Ca, and 65.7 emu/g for uncoated MNPs. MNPs had a marginal effect on the adhesive’s photopolymerization. The mean color difference between G0 and G2 was close to the 50:50% acceptability threshold, whereas the other groups were far apart from G0. The mean whiteness index of G2 did not differ significantly from that of G0; G1 deviated marginally from G0, whereas G3 differed significantly from G0. *Conclusions*: These results suggest that MNP coating can mitigate the influence of MNP-laden dental adhesives on the color of restorations.

## 1. Introduction

Magnetic dental adhesives are obtained by incorporating magnetic nanoparticles (MNPs) into conventional dental adhesives [1,2]. The most common option is to mix commercially available dental adhesives with iron-oxide nanoparticles because of their biocompatibility and excellent magnetic properties [3,4].

Such adhesives were first proposed by Li et al. to control these materials with magnetic forces [1]. In addition to using 2% iron-oxide MNPs, they augmented the adhesive with 5% dimethylaminohexadecyl methacrylate (DMAHDM) and 20% nano-sized amorphous calcium phosphate (NACP). This new material provided a 59% increase in the dentin shear bond strength, presumably because the adhesive was pulled by the magnetic field deeply into the dentinal tubules prior to photopolymerization. MNPs alone did not influence lactic acid production by biofilms formed on the surface of adhesive disks. However, the addition of DMAHDM and NACP brought about a 25-fold reduction in lactic acid production. Thus, colony-forming units in biofilms revealed no influence of MNPs or NACP, while they displayed a reduction by four orders of magnitude due to DMAHDM. Therefore, it was concluded that the augmented adhesive may prevent secondary cavities [1].

The influence of the applied magnetic field on the thickness of an MNP-laden adhesive layer has been investigated by Zaharia et al. [5,6,7]. These works demonstrated that, under the action of a permanent magnet before and during photopolymerization, the adhesive layer becomes thinner as it is pulled into the irregularities of the prepared tooth surface. This conclusion has been supported by optical microscopy, scanning electron microscopy (SEM), and micro-computed tomography (CT) [5].

Recently, Garcia et al. optimized the formulation of an MNP-doped dental adhesive [8], characterized its mechanical performance, visualized its mechanism of action, and ascertained its biocompatibility. Its microtensile bond strength was higher than that of the control adhesive. This finding was consistent with SEM images, which revealed a better mechanical interlock between the exposed dentin-bound collagen and the MNP-doped adhesive compared to the control [8].

Nevertheless, the color of a dental adhesive may change upon incorporating MNPs [8]. This is an important shortcoming, as the appearance of teeth is a serious concern for people from many cultures [9]. A perceptible improvement in dental lightness leads to more positive judgments of four personality traits in women: social competence, intellectual ability, psychological adjustment, and relationship satisfaction [9]. Also, it has been found to boost attractiveness, happiness, social relations, and academic performance [10]. Therefore, it is important to address the esthetic impact of magnetic dental adhesives in correlation with their functional capabilities.

Color science provides dental practitioners with tools for describing tooth color and whiteness [11]. Traditionally, shade guides have been used for color assessment and communication in dentistry. However, visual shade matching using a shade guide is error-prone because it depends on the observer’s training, lighting conditions, and environmental factors that affect the color perception of the observer (e.g., the vivid colors of oral soft tissues, tooth texture and translucency) [12]. Instrumental color assessment and the use of the CIELAB color space have enabled dentists to overcome the limitations of subjective shade matching [13].

In the CIELAB color space [14], a given color is specified by three coordinates: $L^{*}$ (location along the grayscale axis), $a^{*}$ (location along the red–green axis), and $b^{*}$ (location along the yellow–blue axis). These color coordinates are utilized to express perceptual attributes of that color, such as lightness, ranging from black ($L^{*}$ = 0) to white ($L^{*}$ = 100); hue, $h_{ab}=\tan^{-1}\left(b^{*}/a^{*}\right)$; and chroma, $C_{ab}=\sqrt{{a^{*}}^{2}+{b^{*}}^{2}}\;$.

Color differences can be expressed in terms of the distance, $\Delta E_{ab}$, between the corresponding points in the CIELAB color space [11]. Color differences have been used to establish thresholds needed to ensure acceptable esthetics at reasonable costs [15]. The 50:50% perceptibility threshold (PT) is the color difference noticed by half of the observers. Likewise, the 50:50% acceptability threshold (AT) is the color difference deemed acceptable by half of the observers. It would be ideal to keep the color difference between a restoration and the adjacent teeth below the PT, but the high costs of such personalized recipes force dental practitioners to aim for restorations that differ in color from the neighboring teeth by at most the AT. Although color thresholds are commonly expressed in terms of $\Delta E_{ab}$, recent studies urge the use of improved color difference formulas [15], such as the CIEDE2000 formula, $\Delta E_{00}$ [16].

Besides color measurement, a quantitative description of perceived whiteness is desirable in clinical practice, as well as in dental material research and development. Therefore, a variety of whiteness indices have been proposed, among which the CIELAB-based whiteness index for dentistry, $WI_{D}$, has demonstrated the best correlation with the perceptual whiteness of teeth [17].

Iron-oxide nanoparticles are typically black or brown. They can alter the color of a dental adhesive and, consequently, that of the restored tooth. Indeed, impaired esthetics has been reported as a drawback in reinforcing dental adhesives with MNPs [5]. To our knowledge, there are no quantitative studies in the literature regarding the esthetic issues raised by augmenting dental adhesives with MNPs. Therefore, the present study was designed to evaluate the impact of magnetic dental adhesives on the restoration’s color and whiteness. To alleviate their esthetic impact, we proposed coating the MNPs with mineral layers. Our working hypothesis was that the extent of color change induced by the incorporated MNPs can be reduced to a clinically irrelevant level by the proper choice of coating material.

## 2. Materials and Methods

Iron (III) chloride hexahydrate (FeCl_3_ × 6 H_2_O), iron (II) chloride tetrahydrate (FeCl_2_ × 4 H_2_O), ammonia solution (25%), tetraethyl orthosilicate (TEOS, 98%), absolute ethanol, ammonia solution (25%), calcium nitrate tetrahydrate, Ca(NO_3_)_2_ × 4 H_2_O, and sodium hydroxide NaOH were all purchased from Sigma-Aldrich (Burlington, MA, USA) and utilized as received.

### 2.1. Synthesis and Coating of Iron-Oxide Nanoparticles

The functionalized MNPs were prepared by using a multi-step preparation method involving three simple, environmentally friendly, and economically efficient reaction steps.

(1) For the synthesis of Fe_3_O_4_ MNPs with a relatively narrow size distribution of approximately 10 nm in diameter, the co-precipitation method of Fe(II) and Fe(III) salts in a basic medium in an inert atmosphere was used. In a typical experiment, 5.4 g of FeCl_3_ (0.1 M) and 1.99 g of FeCl_2_ (0.05 M), Fe^3+^:Fe^2+^ = 2:1 molar ratio, were dissolved in 200 mL of distilled water. To the so-prepared solution, 200 mL of NH_3_ solution (25%) was added under vigorous stirring at 70 °C under a continuous argon flow. The reaction system was kept at 70 °C for 2 h, and the pH of the solution was controlled to approximately 12 during the formation of the Fe_3_O_4_ nanoparticles. After cooling the system at room temperature, the precipitate was magnetically separated and washed with distilled water to a neutral pH.

(2) For the synthesis of silica-coated nanoparticles (MNPs-SiO_2_), we first chose to coat the surface of the MNPs with a layer of controlled-thickness SiO_2_ shell, which is chemically inert to the action of acids and/or bases and sufficiently resistant to mechanical procedures. This coating can ensure improvements in the chemical and mechanical stability of the final MNPs. The synthesis of the SiO_2_ layer was performed using a modified Stoeber method, by the hydrolysis of the SiO_2_ precursor, i.e., tetraethyl orthosilicate (TEOS), in a basic medium on the surface of the MNPs [18]. Thus, an aqueous solution of ethanol (320 mL) and water (80 mL) (ethanol/water = 4:1 volume ratio) was mixed with 0.4 g (1 wt. %) of MNPs, and the solution was sonicated using an ultrasonic finger for 10 min. Then, the solution was mixed with 8 mL of aqueous ammonia solution (25 wt. %), and 6 mL of TEOS (18 mM) was added dropwise under vigorous magnetic stirring. The reaction was allowed to continue at room temperature for 1 h under magnetic stirring (at 500 rpm). Afterwards, the product was separated using an external magnet and washed several times with distilled water. The final product was collected and dried at 60 °C for 12 h.

(3) For the synthesis of calcium-covered composites (MNPs-Ca), an inorganic Ca-based coating was chosen as the final shell of the MNPs because, in addition to being a major improvement in the color of the final magnetic material, it can act as a good thermal and electrical insulator. Thus, the surface of the functionalized MNPs, with a double layer of SiO_2_ and Ca, is chemically inert. Therefore, it does not interfere with the polymerization reaction of the adhesive and does not affect the degree of conversion of the adhesive. The preparation of the Ca-based inorganic layer on the MNPs’ surface was performed by directly reducing a Ca precursor in a basic solution. For the synthesis of the Ca layer on the surface of the MNPs, 94.5 g of calcium nitrate tetrahydrate, Ca(NO_3_)_2_ × 4 H_2_O, was dissolved in distilled water (8.5 wt.%), and 1 g of MNPs-SiO_2_ was added. A solution of sodium hydroxide, NaOH (16 g, 1.6%), was added dropwise, with an addition rate of 1 mL/min, under strong magnetic stirring (at 1200 rpm) at room temperature. After 3 h of reaction at room temperature, the solution was filtered, and the resulting precipitate was washed 3 times successively with 100 mL of water, then dried in the oven for 1 h at 60 °C.

### 2.2. Characterization of Iron-Oxide Nanoparticles

The morphology of the MNPs at each synthesis step was investigated using transmission electron microscopy (TEM) and scanning electron microscopy (SEM). These analyses were conducted using a Hitachi HD-2700 scanning transmission electron microscope (Hitachi High-Technologies Corporation, Tokyo, Japan), equipped with a cold-field emission gun, operating at an acceleration voltage of 200 kV and designed for a high resolution of 0.144 nm. The magnetic properties of MNPs at room temperature were determined using a vibrating sample magnetometer. After the submission of this work, the preparation and characterization of these MNPs were further detailed [19].

### 2.3. Sample Preparation

**Figure 1:**
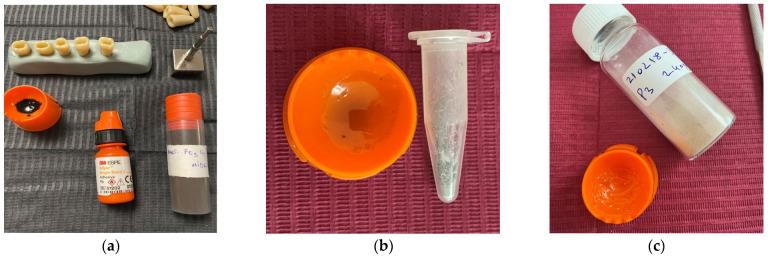
Photos of the steps of the dental adhesive preparation: (**a**) employed materials, (**b**) adhesive loaded with MNPs wrapped in SiO_2_ coating (Group 1), and (**c**) adhesive loaded with MNPs finally covered with Ca-based coating (Group 2).

Four groups, each consisting of 15 artificial tooth specimens, were prepared for this study (Figure 1a). Group 0 (G0) comprised artificial teeth restored using a conventional dental adhesive, Single Bond 2 (3M ESPE, Two Harbors, MN, USA); Group 1 (G1) was processed with the same adhesive loaded with MNPs wrapped in a SiO_2_ coating; Group 2 (G2) was prepared using this adhesive loaded with MNPs covered with both SiO_2_ and a Ca-based coating; and Group 3 (G3) was processed with the same adhesive but loaded with uncoated MNPs. The Single Bond 2 was chosen for this study because it is a widely used dental adhesive that already contains 10% by weight (thus, it is already reinforced) SiO_2_ nanoparticles of 5 nm in diameter. Therefore, we hypothesized that silica-coated MNPs would adhere well to the polymerized adhesive matrix.

**Figure 2:**
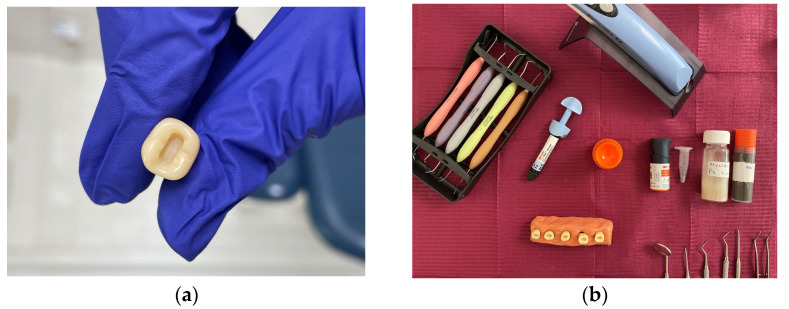
Sample preparation: (**a**) prepared tooth specimen with a Class I cavity and (**b**) armamentarium.

The MNP-laden adhesive was obtained by mixing one curette of MNP powder with one drop of adhesive and manually stirring it for 15 s to obtain a uniform mixture. Then, the artificial tooth specimens were prepared with similar Class I occlusal cavities, using a cylindrical bur on the high-speed instrument under water cooling. The depth of the cavity was 4 mm, and the length was between the marginal crests of the tooth (Figure 2a). The instruments used for sample preparation are shown in Figure 2b.

Different methods were used to prepare the samples of each group, as follows:

Group 0 (control) samples were created using the standard protocol for direct restorations. The etching gel was applied, and after 30 s, the surface was rinsed and dried. Then, the Single Bond 2 adhesive was applied and brushed for 15 s on the surface of the cavity. In order to obtain a thin and uniform layer of adhesive, air was blown for 5 s on the surface of the tooth. The adhesive was light-cured for 10 s, layers of composite were applied, and the composite was then light-cured for 20 s until the cavity was filled.

**Figure 3:**
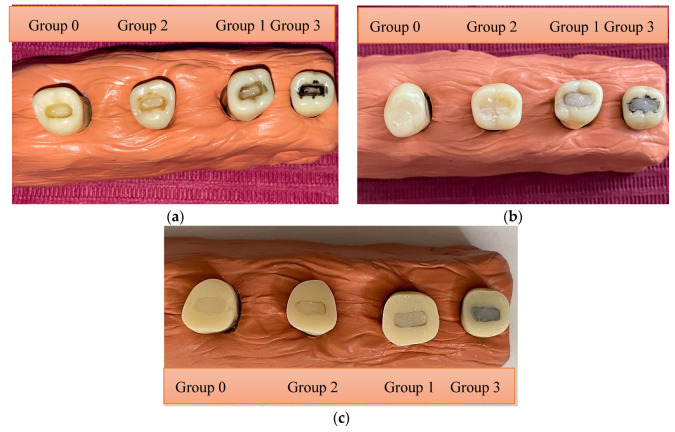
Steps of the sample preparation: (**a**) samples of each group (Group 0 to 3) with the layer of adhesive applied and light cured, (**b**) samples with direct composite restorations, and (**c**) final aspect of the specimens prepared for evaluation. Specimens from Group 2 were placed next to those from Group 0 in order to facilitate a visual comparison. Recall that Group 2 consists of samples prepared with adhesive loaded with MNPs wrapped in a Ca-based coating, whereas Group 0 contains specimens prepared with the same adhesive with no MNPs. Group 1 has SiO_2_-coated MNPs, whereas Group 3 has uncoated MNPs.

For Groups 1, 2, and 3, the working protocol was the following: (1) an etching gel was applied for 30 s, rinsed, and then the surface was dried; (2) the MNP-laden adhesive was brushed onto the surface of the cavity; (3) a permanent magnet was positioned and maintained for 2 min. as close as possible to the surface of the sample in order to attract the MNPs; (4) the adhesive layer was light-cured for 10 s; and finally, (5) layers of composite were applied and light cured for 20 s until the cavity was completely filled (Figure 3a,b).

As a final step, the occlusal surface of each sample was sectioned 2 mm below the tip of the cuspids and polished to obtain a flat surface for color matching—Figure 3c.

### 2.4. Fourier Transform Infrared (FTIR) Spectroscopy for Assessing the Polymerization Grade of Magnetic Dental Adhesives

The polymerization grade (PG) of dental composites is an important parameter indicating the extent of the chemical reaction that converts monomers into polymers during light curing. We quantified PG in terms of the degree of conversion (DC%), which can be calculated using FTIR spectroscopy, as suggested by Morares et al. [20]:

(1) $$\mathrm{DC}\%=\left\{1-\frac{{\left[Abs(\mathrm{aliphatic})/Abs(\mathrm{aromatic})\right]}_{\mathrm{polymer}}}{{\left[Abs(\mathrm{aliphatic})/Abs(\mathrm{aromatic})\right]}_{\mathrm{monomer}}}\right\}\times 100\%$$

where *Abs* denotes the height of the corresponding absorption band. Further on, PG was derived as PG = 1/(1 − DC%/100), obtaining it as a dimensionless value that reflects the completeness of polymerization.

FTIR spectra were recorded for a droplet of a commercial dental adhesive before photopolymerization and for patches of dental adhesives from G0, G1, G2, and G3, light-cured for 10 s and for 20 s, with the latter representing the full duration recommended by the adhesive’s manufacturer.

### 2.5. Objective Assessments of Tooth Color Shades

Tooth color was evaluated using a Vita Easy Shade 4.0 digital spectrophotometer (VITA Zahnfabrik, Bad Säckingen, Germany), a widely used instrument with an accuracy (i.e., the ability to identify the correct color shade) of 92.6% and a reliability (i.e., the ability to consistently provide the same color shade for the same specimen) of 96.5% [13]. The spectrophotometer was calibrated at every start-up and operated in single-point measurement mode. Specimen colors were quantified by their coordinates in the CIELAB color space.

We performed ten successive color shade assessments on the sectioned occlusal surface of each restored artificial tooth (Figure 3c) and computed the arithmetic mean of the recorded color coordinates to characterize the color of the given specimen.

Color differences were expressed as the Euclidean distance between two points from the CIELAB color space [14,15],

(2) $$\Delta E_{ab}=\sqrt{{\left(\Delta L^{*}\right)}^{2}+{\left(\Delta a^{*}\right)}^{2}+{\left(\Delta b^{*}\right)}^{2}}$$

as well as in terms of the CIEDE2000 color difference formula [16],

(3) $$∆E_{00}=\sqrt{{\left(\frac{\Delta L^{\prime }}{K_{L}S_{L}}\right)}^{2}+{\left(\frac{\Delta C^{\prime }}{K_{C}S_{C}}\right)}^{2}+{\left(\frac{\Delta H^{\prime }}{K_{H}S_{H}}\right)}^{2}+R_{T}\left(\frac{\Delta C^{\prime }}{K_{C}S_{C}}\right)\left(\frac{\Delta H^{\prime }}{K_{H}S_{H}}\right)}.$$

which includes corrections that compensate for the non-uniformity of the CIELAB color space. The interpretation of mathematical symbols and the implementation of Equation (2) are provided in the literature [21].

Color differences between pairs of experimental groups (G0-G1, G0-G2, G0-G3, G1-G2, G1-G3, and G2-G3) were expressed, in terms of both $\Delta E_{ab}$ and $\Delta E_{00}$, based on the means of color space coordinates of the specimens from each group. Furthermore, to test whether perceived tooth whiteness was affected by loading dental adhesives with MNPs with various coatings, we computed the CIELAB-based whiteness index developed for the assessment of whiteness in dentistry as follows [17]:

(4) $${WI}_{D}=0.511L^{*}-2.324a^{*}-1.100b^{*}$$

### 2.6. Data Analysis

Experimental results are reported here as mean ± standard deviation (SD). Statistical calculations were performed using MedCalc Version 23.0.9 (MedCalc Software, Ostend, Belgium) and G*Power 3.1.9.7 (The G*Power Team, Düsseldorf, Germany).

The sample size was estimated from preliminary data on the lightness coordinates of 10 specimens from each experimental group (G0, G1, G2, and G3). For G0, the mean value of $L^{*}$ was 77.987 and the corresponding SD was 2.1994; for G2, the mean value was 72.499 and the SD was 7.492, resulting in an effect size, *d* = 0.994. For a statistical power of 0.8 and a significance level of 0.05, the G*Power software provided a sample size of 14. When G1 or G3 was compared to G0, the lightness differences were larger, leading to larger effect sizes and smaller sample-size estimates. Therefore, we decided to supplement each experimental group with 5 additional specimens, reaching a sample size of 15 specimens/group.

We used the Shapiro–Wilk test to evaluate the normality of the recorded color coordinates within each group. For normally distributed data, we applied the one-way analysis of variance (ANOVA), followed by Scheffé’s procedure for pairwise comparisons to identify statistically significant differences between the mean color coordinates of teeth restored using different adhesives. For data that deviated from normality, we performed the Kruskal–Wallis test to identify significant differences between groups. The level of statistical significance was set to *p* < 0.05.

The colors of specimens from different groups were compared by conducting a Bland–Altman analysis. It is based on plotting differences vs. means of pairs of experimental values provided by different methods (e.g., color coordinates of specimens created using different adhesives) [22,23].

## 3. Results

### 3.1. Morphological Characterization of MNPs with Different Coatings

**Figure 4:**
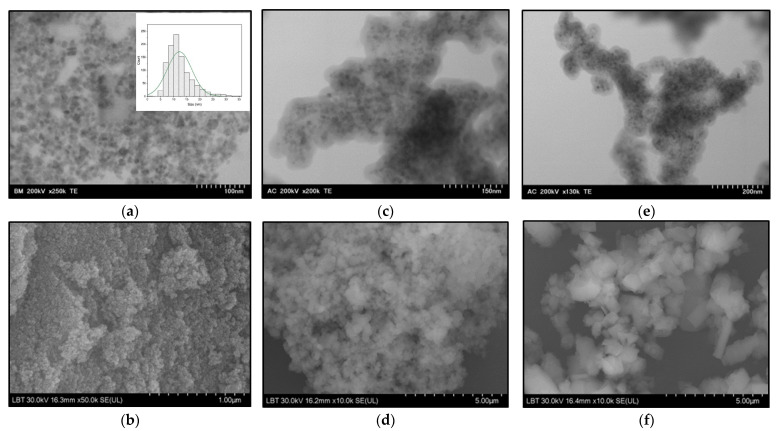
TEM and SEM images of (**a**,**b**) Fe_3_O_4_ MNPs, (**c**,**d**) silica-coated nanoparticles (MNPs-SiO_2_), and (**e**,**f**) magnetic composites with Ca-based coating (MNPs-Ca).

The morphology of the Fe_3_O_4_ MNPs, silica-coated MNPs (MNPs-SiO_2_), and magnetic nanocomposites covered by Ca(OH)_2_ (MNPs-Ca) are shown using TEM and SEM images in Figure 4. A uniform, well-dispersed nanoparticle structure with an average diameter of 12 nm is observed in these images that were recorded for the uncoated MNPs (Figure 4a,b). The reported mean size was obtained via size distributions deduced from the TEM analysis. Figure 4c,d shows the MNPs embedded in the SiO_2_ layer (MNPs-SiO_2_), and a slight aggregation of the nanoparticles is observed during the SiO_2_ coating process. Figure 4e,f shows the MNPs embedded in a Ca-based inorganic layer (MNPs-Ca). A relatively uniform distribution of MNPs in the inorganic layer can be observed, and the structural change from the globular shape, given by the SiO_2_ structure, to the cubic shape, given by the Ca(OH)_2_ structure, is visible. The thicknesses of the two inorganic layers covering the MNPs were calculated, also from TEM images; the average thickness of the SiO_2_ layer was 73.1 nm ± 9.9 nm (n = 17), and the average thickness of the Ca(OH)_2_ layer was 19.97 nm ± 2.27 nm (n = 17).

**Figure 5:**
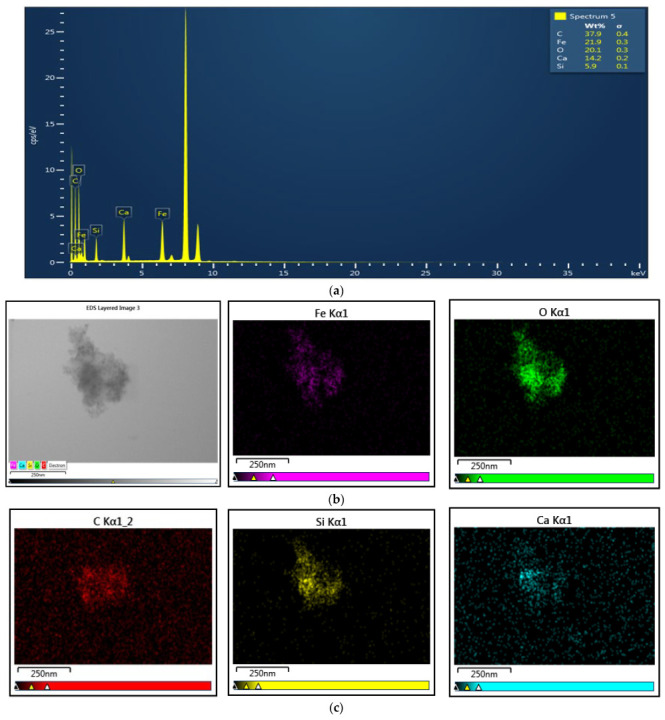
(**a**) EDX spectrum and (**b**,**c**) EDX elemental mapping of a representative sample of MNPs covered with a double layer of SiO_2_ and Ca.

The chemical characterization (i.e., elemental analysis) of dental materials was determined using energy-dispersive X-ray spectroscopy (EDX). Figure 5a shows the EDX spectrum for a representative sample of magnetic nanocomposites. The EDX spectrum shows the constituent elements of each component—the MNPs (Fe, O), the SiO_2_ layer (C, Si, O), and the calcium-based coating (Ca, O)—as well as the atomic concentrations of all these elements. Figure 5b shows the EDX elemental mapping, representing the spatial distribution of each element, in a representative sample of magnetic nanocomposite. A uniform distribution of each element within the sample was observed, suggesting a good coverage of the nanoparticles with homogeneous and continuous layers of SiO_2_, as well as a Ca-based inorganic layer.

### 3.2. Magnetic Properties of the Synthesized Iron-Oxide Nanoparticles

**Figure 6:**
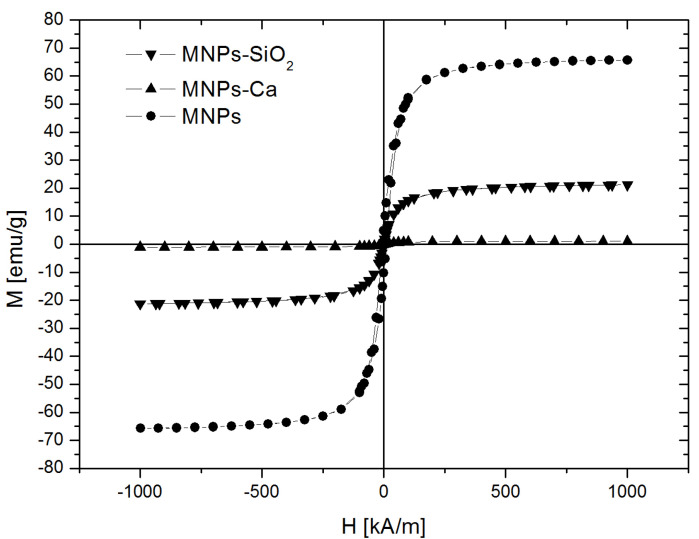
Magnetic hysteresis loops measured at room temperature for the Fe_3_O_4_ MNPs, silica-coated nanoparticles (MNPs-SiO_2_), and Ca-covered magnetic composites (MNPs-Ca).

To ensure that this material is applicable in dental restorations and can be applied to the surface of dental cavities under the effects of an external magnetic field, the material must have suitable magnetic properties. Figure 6 shows the magnetization curves recorded at room temperature for Fe_3_O_4_ MNPs, silica-coated nanoparticles (MNPs-SiO2), and Ca-covered magnetic nanocomposites (MNPs-Ca). The saturation magnetization value was 65.7 emu/g for the uncoated MNPs, 21.3 emu/g for MNPs-SiO2, and 1 emu/g for MNPs-Ca. The decrease in magnetization value was expected because the coating of the MNPs consists of non-magnetic layers. However, the saturation magnetization remained sufficiently high for the intended application.

### 3.3. Evaluation of the Photopolymerization of Dental Adhesives Loaded with MNPs

**Figure 7:**
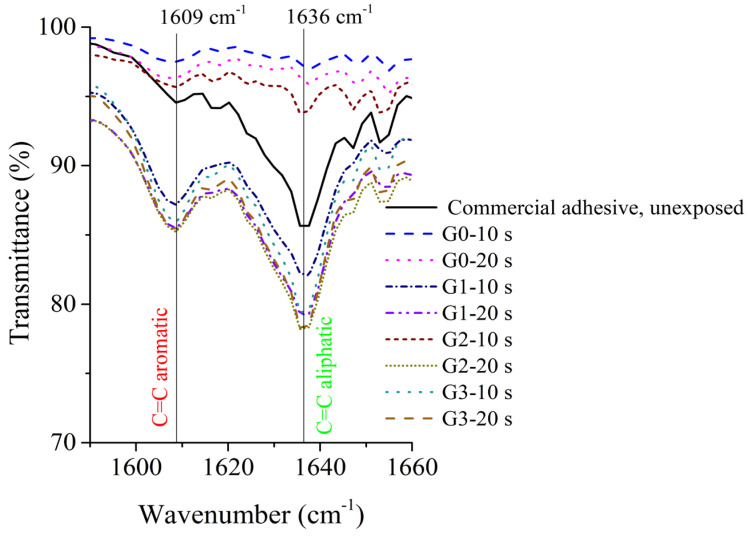
FTIR spectra recorded at room temperature for dental adhesive samples (G0, G1, G2, and G3) at various UV exposure times. The peaks corresponding to C=C aromatic and C=C aliphatic bonds are shown; these were utilized to determine the degree of conversion (DC%) in dental adhesive samples according to Equation (1).

We utilized FTIR spectroscopy to evaluate the PGs for the different groups of samples (i.e., G0, G1, G2, and G3) exposed to UV curing for varying periods of time (specifically, 10 s and 20 s). Figure 7 presents the corresponding FTIR spectra. The PGs, calculated from DC% values given by Equation (1), are listed in Table 1, highlighting the impact of curing duration and material composition on polymerization efficiency.

### 3.4. Color Shades of Occlusal Cavities Restored Using Dental Adhesives with Different MNP Content

According to the Shapiro–Wilk test, the color coordinates of specimens from each group were normally distributed (*p* > 0.05), except for the lightness coordinates of Group 2 (*p* = 0.004). All the corresponding *p*-values are listed in the Supplementary Materials, Table S1.

The ANOVA test revealed that there are statistically significant differences between the mean values of $a^{*}$ and $b^{*}$ of the samples from the four experimental groups. The Seffeé test revealed significant differences between the mean color coordinates of the following pairs: G0-G1, G0-G3, G1-G3, and G2-G3 (Tables S2 and S3). The same conclusion was conveyed by the Kruskal–Wallis test applied for $L^{*}$ (Table S4).

**Figure 8:**
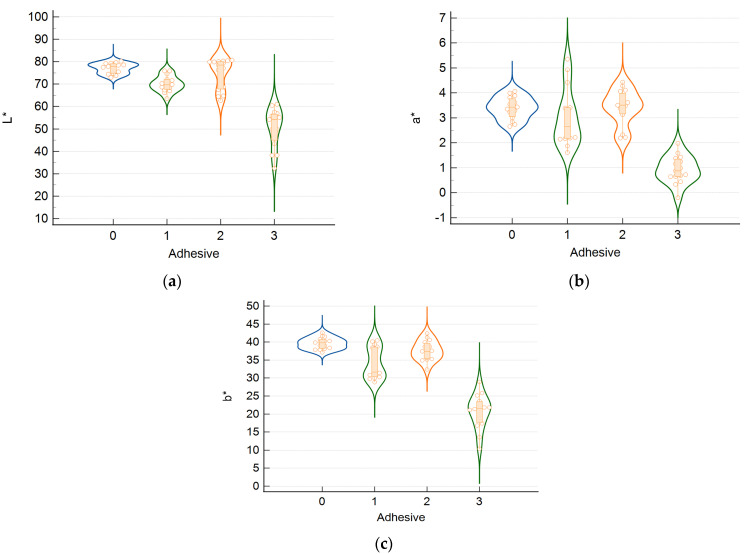
Violin plots of color space coordinates of restored occlusal cavities. Specimens were prepared using a conventional dental adhesive containing no MNPs (0), MNPs with SiO_2_ coating (1), MNPs with Ca-based coating (2), or uncoated MNPs (3). (**a**) Plots of the color coordinate L* associated with lightness (black–white axis). (**b**) Plots of the a* coordinate (red–green axis). (**c**) Plots of the b* coordinate (yellow–blue axis). The width of a violin plot represents the probability density function—values corresponding to wide regions occur more frequently in the data set. The rectangle in the middle of a violin plot spans the interquartile range (the middle half of the data set), whereas the horizontal segment that divides the rectangle represents the median.

Figure 8 shows the violin plots of the CIELAB color space coordinates of all the specimens prepared for this study. They indicate that the MNPs within the magnetic dental adhesives did affect the distribution of the color coordinates of restored teeth. With a single exception ($L^{*}$ for G1), the spread of the data is larger for the samples from G1, G2, and G3, prepared with magnetic dental adhesives.

**Figure 9:**
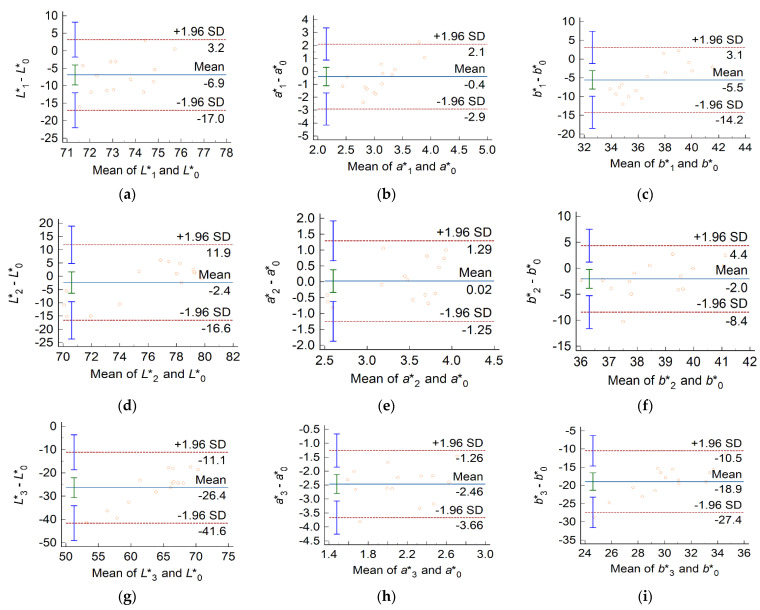
Bland–Altman analysis of differences between color space coordinates of specimens prepared using dental adhesives with different MNP content. The index 0 designates a commercial dental adhesive, whereas indices 1, 2, and 3 refer to the same adhesive loaded with MNPs with SiO_2_ coating, Ca-based coating, and no coating, respectively. (**a**) $L_{1}^{*}$ compared with $L_{0}^{*}$, (**b**) $a_{1}^{*}$ compared with $a_{0}^{*}$, (**c**) $b_{1}^{*}$ compared with $b_{0}^{*}$, (**d**) $L_{2}^{*}$ compared with $L_{0}^{*}$, (**e**) $a_{2}^{*}$ compared with $a_{0}^{*}$, (**f**) $b_{2}^{*}$ compared with $b_{0}^{*}$, (**g**) $L_{3}^{*}$ compared with $L_{0}^{*}$, (**h**) $a_{3}^{*}$ compared with $a_{0}^{*}$, and (**i**) $b_{3}^{*}$ compared with $b_{0}^{*}$. In each panel, the solid horizontal line, labeled “Mean”, represents the bias, defined as the mean of the differences between pairs of values. Dashed lines delimit the 95% interval of agreement, which contains about 95% of the markers; here, SD stands for the standard deviation of differences. The vertical segments centered on the horizontal lines represent the 95% confidence interval of the corresponding statistical parameter.

In Figure 9, each Bland–Altman plot represents the differences between the means of the color coordinates of restorations performed using an MNP-loaded adhesive and those based on the MNP-free adhesive. In such a plot, each marker corresponds to a pair of teeth restored using different adhesives but with the same fluid composite material. Uncoated Fe_3_O_4_ nanoparticles had a major impact on all color coordinates, indicated by the large, negative biases in both $L^{*}$ and $b^{*}$ (Figure 9g,i). Relatively large biases of the same sign were also observed in Figure 9a,c, indicating that the SiO_2_ coating was not fully effective in masking the dark color of the MNPs. By contrast, the Ca-based coating rendered biases close to zero (Figure 9d,f). However, the relatively wide intervals of agreement suggest that differences between individual pairs might still be noticeable.

**Figure 10:**
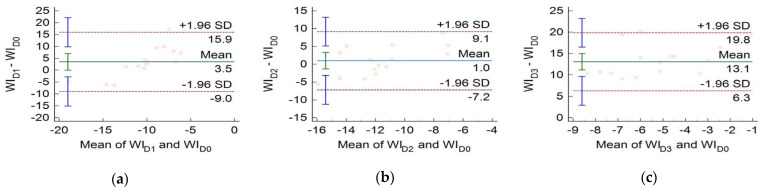
Bland–Altman plots of differences vs. means of the whiteness indices of teeth restored using adhesives augmented with MNPs of different coatings compared to teeth restored using the same adhesive without incorporated MNPs. (**a**) Comparison of samples from groups G1 and G0, (**b**) comparison of G2 and G0, and (**c**) comparison of G3 and G0.

The color differences computed from the mean color coordinates of the four groups are listed in Table 2. The pairwise color differences computed from the mean color coordinates of the specimens that compose the four experimental groups exceed the 50:50% AT values reported in the literature [24]. However, the G0-G2 pair is promising because for this pair, both the $\Delta E_{ab}$ and $\Delta E_{00}$ values are at the verge of the acceptability thresholds. Finally, the influence of nanoparticle coating on the CIELAB whiteness index of the restored teeth is illustrated in the Bland–Altman plots in Figure 10.

The whiteness index, ${\mathrm{WI}}_{D}$, is given by Equation (4) [17]. Figure 10a indicates that, on average, tooth whiteness was marginally affected by the adhesive augmented with MNPs-SiO_2_, whereas MNPs-Ca had no significant impact. Indeed, zero is contained in the 95% CI of the mean difference in Figure 10b. In contrast, bare iron-oxide nanoparticles caused a significant change in ${\mathrm{WI}}_{D}$ (Figure 10c).

## 4. Discussion

This study aimed to evaluate the impact of magnetic dental adhesives on the color of restored teeth. We created occlusal cavities in artificial teeth and restored them using a commercial dental adhesive, as well as the same adhesive augmented with iron-oxide nanoparticles with diverse coatings.

Observing the sizes of the MNPs and the thicknesses of the SiO_2_ and Ca(OH)_2_ layers, we can conclude that the prepared magnetic composites possess appropriate properties for dental applications. The magnetic particle size is suitable for ensuring adequate magnetic properties, the SiO_2_ layer is thick enough to provide mechanical and chemical strength, and the Ca(OH)_2_ layer masks the dark color of the iron oxide. Based on the morphological characterization of the prepared MNPs, it can be claimed that magnetic nanocomposites are suitable for dental applications in terms of the size and thickness of inorganic layers, as demonstrated in [5,6,7]. The decrease in saturation magnetization is explained by the coating of the MNPs with non-magnetic layers. Nevertheless, neither of the two coating layers was too thick to impair the magnetic properties of the nanocomposites.

FTIR spectroscopy was used to characterize the photopolymerization of augmented adhesive samples. G0 had the highest PG values across all time intervals, indicating superior polymerization kinetics, possibly due to better resin composition or initiator efficiency. G1, G2, and G3 exhibited lower and more consistent PG values, presumably because of different filler content that impacts their polymerization behavior. For G2 and G3, the PG stabilizes at approximately 1.8 at 10–20 s, potentially indicating a saturation point where further UV exposure does not significantly enhance the polymerization. The differences between samples may have stemmed from (1) resin components, (2) filler content and dispersion, and (3) initiator efficiency (as samples with optimized photoinitiators are more likely to achieve a higher PG). The results are critical for clinical applications, as higher PG values generally correlate with improved mechanical properties, lower residual monomers, and enhanced biocompatibility. However, overexposure to UV light should be balanced against potential thermal effects and material degradation. Further research is warranted to test the mechanical properties of magnetic dental adhesives in conjunction with their photopolymerization kinetics.

The color and perceptual whiteness of the restored teeth were quantified using a digital spectrophotometer and analyzed within the CIELAB color space. The results confirmed our working hypothesis, that nanoparticle coating can alleviate the esthetic issues raised by reinforcing dental adhesives with iron-oxide nanoparticles.

The color assessments reported in this study can be interpreted in terms of several visual thresholds established for dentistry [24]. In terms of $\Delta E_{ab}$, the just-noticeable difference, or the 50:50% perceptibility threshold (PT), was 1.2 [25]. Using the CIEDE2000 color difference formula, the PT was determined as $\Delta E_{00}$ = 0.8 [25]. The mean color differences recorded in our study exceeded all these PT values (Table 1). This indicates that, despite the MNP coatings, magnetic dental adhesives bring about a noticeable change in the color of restorations. The question is whether the observed color differences would be deemed acceptable by dentists and patients. The AT, expressed via $\Delta E_{ab}$, was 2.7 in the study by Paravina et al. [25] and 3.5 in the work of Ghinea et al. [26]. Using the CIEDE2000 formula, the AT was established as $\Delta E_{00}$ = 1.9 [27] or $\Delta E_{00}$ = 1.8 [25]. These values suggest that the mean color difference between Groups 0 and 2 is clinically acceptable.

Besides color difference formulas, another option to tackle the question of whether restorations prepared with magnetic dental adhesives differ in appearance from conventionally prepared ones is to compute differences among the $L^{*}$, $a^{*}$, and $b^{*}$ corresponding to different groups and to compare them with the AT values determined by Lindsey and Wee [28]: $\Delta L^{*}$= 1.0, $\Delta a^{*}$= 1.0, and $\Delta b^{*}$= 2.6. According to these criteria, the mean differences calculated for $L^{*}$ and $b^{*}$ exceed the AT thresholds. In light of the most extensive, multicentric study of visual thresholds for dentistry [26], even the best-matching groups are marginally beyond the AT.

The Bland–Altman analysis in Figure 9 demonstrates that individual differences between CIELAB color coordinates are larger than the mean differences, suggesting that specific pairs of samples considered from groups G0 and G2 most likely differ in color beyond the clinically acceptable limit. Sources of variability include inappropriate mixing and differences in the thickness of coating layers. The elongated distributions of the color coordinates of MNP-laden samples are indicative of a less homogeneous spatial arrangement of pigments within augmented adhesives compared to conventional ones (Figure 8). However, a scrutiny of group medians suggests that Group 2 might be satisfactory from the esthetic point of view. Indeed, in the CIELAB color space, the points corresponding to individual specimens from Group 2 are mainly colocalized with those from Group 0 (as presented in the Supplementary Materials, Figure S1). Further research is warranted to ensure consistent color control at the individual level. Better coating materials are needed, along with improved control of coating-layer thickness.

Thus, considered together, the above results confirm our working hypothesis, but future work is needed to address sample heterogeneity and explore further coating options.

The benefits of using iron-oxide nanocomposites with core–shell architecture may extend beyond the restoration’s color control. While bare iron-oxide nanoparticles did not confer significant antibacterial activity to the dental adhesives that incorporated them [1,8], Mokeem et al. suggested coating superparamagnetic iron-oxide nanoparticles with a shell loaded with antibacterial compounds [29,30]. Such an approach would preserve magnetic responsiveness while boosting antibacterial properties with a time course controlled by smart drug delivery techniques. The need for a prolonged release via the core–shell approach was reiterated in a study of dental adhesives doped with benzyldimethyldodecyl ammonium chloride (BDMDAC), which is an antiseptic [31]. The BDMDAC addition did not affect the adhesive’s photopolymerization and mechanical properties. In concentrations of 4 to 5% by weight, BDMDAC was highly effective in preventing biofilm formation and had no cytotoxic effects on human gingival fibroblasts. The antibacterial activity, however, faded away during artificial aging (i.e., 104 temperature cycles in artificial saliva) that is equivalent to one year of clinical use. The authors attributed this decline to the leaching of the antibacterial agent. BDMDAC lacks methacrylate groups. Thus, due to light curing, it is entrapped in the polymer network without being chemically bound to it. A potential solution to this problem is to engineer micro- or nano-structures (e.g., core–shell nanoparticles) capable of a controlled release of BDMDAC [30,31].

A potential issue that must be discussed refers to the progressive decrease in saturation magnetization when the MNPs received the SiO_2_ coating and subsequently the Ca-based coating. Figure 6 suggests that the saturation magnetization still allows for Ca-coated MNPs to fulfill their primary purpose of entering the dentine tubules and thus improving the quality of the dental adhesive. However, one can notice that there is a clear trade-off between magnetic and esthetic properties. Therefore, the less opaque SiO_2_ coating might be considered if higher magnetization saturation proves necessary, although it does not have the best esthetic properties. This trade-off must be considered for all future studies, as well, when testing other, potentially better coatings, to preserve the primary function of the coated MNPs.

To our knowledge, this is the first study to provide a quantitative assessment of the esthetic impact of MNPs incorporated in dental adhesives. It confirms the hypothesis that nanoparticle coating is a feasible strategy to adjust the color of magnetic dental adhesives according to clinical requirements.

The limitations of this study include the following features: employing artificial teeth instead of extracted teeth, using specimens of merely one color shade, investigating a single restoration architecture (i.e., occlusal cavity), and using a dental spectrophotometer for color assessment. Focusing on artificial teeth enabled us to circumvent biological variability and allowed for a proper sample size. Investigating only one class of cavities further limited variability associated with tooth morphology. However, the use of artificial teeth precluded us from assessing the reduction in adhesive layer thickness and the corresponding change in bond strength, since dentinal tubules were absent. Therefore, our results cannot be readily applied in clinical practice. Future research may elucidate whether the coating developed in this study remains appropriate for natural teeth of different color shades, as well. Extracted teeth are planned to be used for the measurement of the mechanical properties of dental adhesives loaded with coated MNPs, as well as for the visualization of the adhesive–dentin interface.

The spectrophotometer employed in this study is commonly used in dentistry. Nevertheless, chromatic differences also exist between different regions of a given sample. For example, in certain images from Figure 3c, the adhesive layer can be distinguished from the rest of the restoration. Since the optical sensor of the spectrophotometer is large compared to the adhesive layer thickness of 10–100 µm, the current methodology was unable to reveal color differences between various adhesive layers and the bulk of the composite filling. Future work may address this problem by employing digital photocolorimetry [32].

The clinical translation of the results reported in this paper hinges on further investigations. Besides toxicity tests and mechanical evaluation, MNP-laden dental adhesives should be tested against physicochemical factors present in the oral environment. Although most MNPs are expected to remain embedded in the adhesive layer, mechanical erosion and microleakage can expose them to saliva and oral bacteria. The long-term stability of the coating should be evaluated in an artificial environment that simulates these circumstances.

## 5. Conclusions

This study evaluated the esthetic benefits of nanoparticle coatings when using dental adhesives augmented with iron-oxide nanoparticles. It demonstrated that certain coating materials can mask the dark color of MNPs, thereby preventing nanoparticles from spoiling the color of the restored tooth. Thus, the present work fills a gap in the literature concerning magnetic dental adhesives. Our results suggest that MNPs wrapped in a double layer of silica and calcium hydroxide can be incorporated into conventional dental adhesives to make them magnetically responsive and esthetically acceptable.

However, further research is needed to mitigate the heterogeneity of the color of restorations prepared using MNP-laden adhesives and to evaluate the mechanical properties of magnetic dental adhesives prepared with MNPs of different coatings. The trade-off between magnetic and esthetic properties must be considered in future studies to ascertain that the coated MNPs preserve their primary function of boosting the force of adhesion and preventing microleakage. Also, the long-term degradation of the coating material in the oral environment is an interesting topic for future research. Following their physicochemical characterization, magnetic dental adhesives should be tested in vitro and in vivo to determine their safety and efficacy. In vitro tests can include cell culture studies and antibacterial assays, while in vivo tests may include animal studies and, eventually, clinical trials.

## Figures and Tables

**Table 1:** Polymerization grade (PG) as a function of the UV exposure time, determined for dental adhesives from groups G0, G1, G2, and G3.

Time of UV Exposure (s)	Mean Value of the PG of Samples			
	G0	G1	G2	G3
10	2.62	1.94	1.90	1.83
20	2.82	1.87	1.80	1.80

**Table 2:** Mean color differences between the four groups of teeth restored using different adhesives (Group 0—with no MNPs, Group 1—with MNPs with SiO_2_ coating, Group 2—with MNPs with Ca-based coating, and Group 3—with bare MNPs, i.e., with no coatings).

Pair	Group 0–Group 1	Group 0–Group 2	Group 0–Group 3	Group 1–Group 2	Group 1–Group 3	Group 2–Group 3
$\Delta E_{ab}$	8.87	3.13	32.56	5.75	23.72	29.46
$\Delta E_{00}$	5.54	1.88	23.47	3.67	18.09	21.67

## Data Availability

The data generated throughout this study are available in the Supplementary Materials. Further information can be obtained from the first author.

## Supplementary Materials

The following supporting information can be downloaded at https://www.mdpi.com/article/10.3390/medicina61071289/s1: Figure S1: Colors of the restored artificial tooth specimens represented in the CIELAB color space; Table S1: *p*-values returned by the Shapiro–Wilk test for color coordinates; Table S2: Results of the one-way ANOVA test for $a^{*}$; Table S3: Results of the one-way ANOVA test for $b^{*}$; and Table S4: The output of the Kruskal–Wallis test for $L^{*}$.
