# Supplementary material for: The Impact of Nanoparticle Coatings on the Color of Teeth Restored Using Dental Adhesives Augmented with Magnetic Nanoparticles
Source: Medicina (Kaunas). 2025 Jul 17;61(7):1289. doi: 10.3390/medicina61071289 (PMC12300422; doi:10.3390/medicina61071289)
Supplement: Supplementary file 1 [file medicina-61-01289-s001.zip › medicina-3668010-supplementary.pdf]

medicina

Supplementary Materials for the Article

Dental adhesives augmented with magnetic nanoparticles: the impact of nanoparticle coating on the color of the restoration

Carina Sonia Neagu, Andreea Codruta Novac, Cristian Zaharia, Meda-Lavinia Negrutiu, Izabell Craciunescu, Vlad Mircea Socoliuc, Catalin Nicolae Marin, Ionela-Amalia Bradu, Luminita Maria Nica, Marius Stef, Virgil-Florin Duma, Mihai Romînu, Cosmin Sinescu

This file presents supplementary information regarding the study reported in the corresponding article. The titles and captions of the enclosed illustrations are supposed to help the reader to understand them as stand-alone items. Their interpretation, however, is only discussed in the main text of the article.

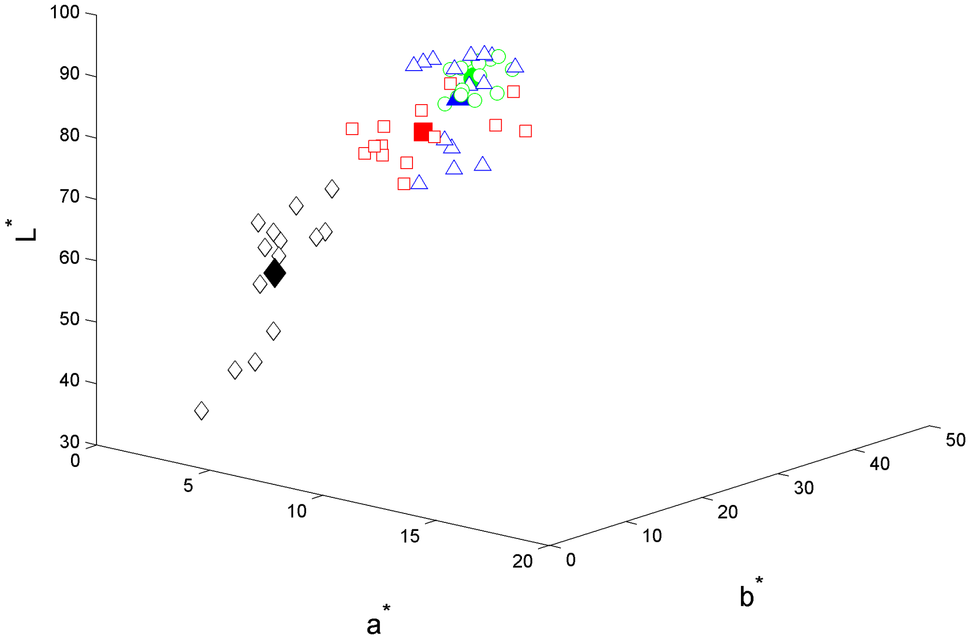

Figure S1. Colors of the restored artificial tooth specimens represented in the CIELAB color space: Group 0 – green circles, Group 1 – red squares, Group 2 – blue triangles, Group 3 – black diamonds. Small, empty markers depict the colors of individual samples, whereas large, solid markers of the same shape stand for the mean of the respective group. (Group 0 – conventional adhesive, Group 1 – adhesive with MNPs of SiO_2 coating, Group 2 – adhesive with MNPs of calcium-based coating, Group 3 – adhesive with bare MNPs).

Table S1. *P*-values returned by the Shapiro-Wilk test for color coordinates. This test evaluates the normality of the distribution of color coordinates determined for different groups of restored teeth.

Group	L*	a*	b*
0	0.2505	0.6649	0.8134
1	0.8705	0.0704	0.0234
2	0.0019	0.1489	0.9702
3	0.0667	0.9979	0.7366

Table S2. Results of the one-way ANOVA test for a^*.

Source of variation	Sum of Squares	DF	Mean Square
Between groups (influence factor)	63.1304	3	21.0435
Within groups (other fluctuations)	32.2671	56	0.5762
Total	95.3975	59	

F-ratio	36.521
Significance level	P < 0.001

Scheffé test for all pairwise comparisons

Group	n	Mean	SD	Different (P<0.05) from group nr.
0	15	3.3947	0.4440	3
1	15	2.9960	1.1395	3
2	15	3.4167	0.7114	3
3	15	0.9320	0.5505	0, 1, 2

Residuals

Shapiro-Wilk test for Normal distribution	W=0.9665 accept Normality (P=0.0982)

Table S3. Results of the one-way ANOVA test for b^*

Source of variation	Sum of Squares	DF	Mean Square
Between groups (influence factor)	3262.4592	3	1087.4864
Within groups (other fluctuations)	723.5812	56	12.9211
Total	3986.0404	59	

F-ratio	84.164
Significance level	P < 0.001

Scheffé test for all pairwise comparisons

Group	n	Mean	SD	Different (P<0.05) from group nr.
0	15	39.6360	1.5931	1, 3
1	15	34.0933	4.3208	0, 3
2	15	37.5960	2.7327	3
3	15	20.7060	4.7968	0, 1, 2

Residuals

Shapiro-Wilk test for Normal distribution	W=0.9901 accept Normality (P=0.9108)

Table S4. The output of the Kruskal-Wallis test for L^*.

Test statistic	40.3562
Corrected for ties Ht	40.3573
Degrees of Freedom (DF)	3
Significance level	P < 0.000001

Post-hoc analysis (Conover)

Group	n	Average Rank	Different (P<0.05) from group nr.
0	15	44.00	1, 3
1	15	28.23	0, 2, 3
2	15	41.77	1, 3
3	15	8.00	0, 1, 2
